# Similar Gene Estimates from Circular and Linear Standards in Quantitative PCR Analyses Using the Prokaryotic 16S rRNA Gene as a Model

**DOI:** 10.1371/journal.pone.0051931

**Published:** 2012-12-19

**Authors:** Athenia L. Oldham, Kathleen E. Duncan

**Affiliations:** 1 The Department of Microbiology and Plant Biology, University of Oklahoma, Norman, Oklahoma, United States of America; 2 The Institute for Energy and the Environment, University of Oklahoma, Norman, Oklahoma, United States of America; Missouri University of Science and Technology, United States of America

## Abstract

Quantitative PCR (qPCR) is one of the most widely used tools for quantifying absolute numbers of microbial gene copies in test samples. A recent publication showed that circular plasmid DNA standards grossly overestimated numbers of a target gene by as much as 8-fold in a eukaryotic system using quantitative PCR (qPCR) analysis. Overestimation of microbial numbers is a serious concern in industrial settings where qPCR estimates form the basis for quality control or mitigation decisions. Unlike eukaryotes, bacteria and archaea most commonly have circular genomes and plasmids and therefore may not be subject to the same levels of overestimation. Therefore, the feasibility of using circular DNA plasmids as standards for 16S rRNA gene estimates was assayed using these two prokaryotic systems, with the practical advantage being rapid standard preparation for ongoing qPCR analyses. Full-length 16S rRNA gene sequences from *Thermovirga lienii* and *Archaeoglobus fulgidus* were cloned and used to generate standards for bacterial and archaeal qPCR reactions, respectively. Estimates of 16S rRNA gene copies were made based on circular and linearized DNA conformations using two genomes from each domain: *Desulfovibrio vulgaris, Pseudomonas aeruginosa*, *Archaeoglobus fulgidus,* and *Methanocaldocococcus jannaschii*. The ratio of estimated to predicted 16S rRNA gene copies ranged from 0.5 to 2.2-fold in bacterial systems and 0.5 to 1.0-fold in archaeal systems, demonstrating that circular plasmid standards did not lead to the gross over-estimates previously reported for eukaryotic systems.

## Introduction

Quantitative PCR (qPCR) is a sensitive and reliable method used to quantify the number of target gene copies in a given sample. The accuracy of absolute quantification relies on the use of standards of known copy numbers run in the same experiment as the sample(s) being analyzed [1]. In environmental and industrial microbiology, microbial counts can be rapidly deduced using molecular methods based on known numbers of 16S rRNA genes or specific functional genes present in the genome [2]. The 16S rRNA gene is the most widely used tool for assessing microbial diversity and numbers in environmental samples using PCR-based methods [1], [3]. The ∼1500 base pair (bp) full-length 16S rRNA gene sequence contains conserved regions that are flanked by hyper-variable regions, and it is the degree of variation within these hyper-variable regions that distinguishes between microbial taxa at various classification levels [4], [5]. Therefore, taxa-specific 16S rRNA gene primer sets coupled with SYBR chemistry is the most cost-effective method to target and quantify the maximum number of microbes at a given classification level within a test sample reviewed in [6].

A recent report has shown that supercoiled plasmid DNA used to generate standard curves grossly overestimated the number of *pcna* gene copies by 8-fold using the microalgae eukaryotic system [7]. Lin et al. [8] reported a similar finding, with a 3-fold increase in the NK603/*zSSIIb* gene(s) using the eukaryotic system, maize. Interestingly, little attention has been paid to the effect of circular plasmid standards in bacterial and archaeal systems which commonly have genomes and plasmids that are circular, although linear forms are found in some cases [9]. In industrial microbiology, 16S rRNA gene copies can be reported as a means of assessing the microbial abundance in a given sample [10] **,** with the caveat that 16S rRNA gene numbers can vary by a log-fold per genome between different species [11]; so if this inherent variation is further amplified by as much as a log-fold due to overestimation by a circular standard, this could have important ramifications for the quantification of microbes of interest in many different industrial and medical settings. Therefore, the goal of this study was to test the feasibility of using a circular plasmid standard purified from transformed bacterial cells with no further preparation for 16S rRNA gene copy number estimates in bacterial and archaeal systems. We hypothesized that circular plasmids would yield similar gene estimates as their linearized counterparts and could therefore be used in lieu of, with the major advantage of minimal standard preparation for continual qPCR analyses. To test this hypothesis, gene estimates based on two circular plasmid standards (supercoiled and nicked circles) were compared to those of two linear standards, a *SpeI*-digested plasmid and a PCR amplicon, using two sets of taxa-specific 16S rRNA gene primers. One set of primers targeted the bacterial 16S rRNA gene while the other set targeted the archaeal 16S rRNA gene. The ratio of estimated to predicted 16S rRNA gene copies were analyzed using sequenced bacterial and archaeal genomes and results presented here demonstrated that circular plasmids did not lead to gross overestimates in 16S rRNA gene copies. Therefore, propagated plasmids suffice for prokaryotic 16S rRNA gene estimates and require less preparation than linearized or PCR-amplicon DNA for use as qPCR standards.

## Methods

### Genomic DNA Preparations

Three bacterial and two archaeal strains, whose genomes had been completely sequenced, were chosen for this study. A freeze-dried culture of the *Thermovirga lienii* type strain Cas60314 (DSM 17291/ATCC BAA-1197) was purchased from the Leibniz Institute-German Collection of Microorganisms and Cell Cultures (DSMZ) and cultured according to manufacturer’s instructions. Genomic DNA was extracted from *T. lienii* using the PowerSoil® DNA isolation kit (MO BIO Laboratories Inc., Carlsbad, CA, USA) according to manufacturer’s instructions and eluted into RT-PCR grade water (Life Technologies, Carlsbad, CA, USA). Lyophilized genomic DNA samples from *Desulfovibrio vulgaris* subsp. *vulgaris* strain Hildenborough (NCIB 8303/ATCC 29579), *Pseudomonas aeruginosa* strain PAO1-LAC (ATCC 47085), *Archaeoglobus fulgidus* strain VC16 (DSM 4304/ATCC 49203), and *Methanocaldococcus jannaschii* strain JAL-1 (DSM 2661/ATCC 43067) were purchased from the American Type Culture Collection (ATCC). The lyophilized samples were reconstituted with RT-PCR grade water (Life Technologies) and DNA concentrations were measured in triplicate using a Qubit® 2.0 fluorometer (Life Technologies) with dsDNA BR Regents according to manufacturer’s instructions. Genomic DNA samples were stored at −20°C until use.

### Cloning of an Archaeal and Bacterial 16S rRNA Gene to Generate Plasmid and Amplicon-based Standards

Representative full-length bacterial and archaeal 16S rRNA gene sequences were amplified from *T. lienii* and *A. fulgidus*, respectively. Sequences from these two strains were chosen as they were the most abundant bacterial and archaeal 16S rRNA sequences found in Alaskan North Slope oil facility samples that our research group monitors [12]. Briefly, 1 µl of genomic DNA was amplified in 25 µl reactions containing 0.625 U of DreamTaq™ polymerase (Fermentas, Glen Burnie, Maryland, USA), 0.2 mM dNTPs (Fermentas), 0.5 M Betaine (Sigma-Aldrich, St. Louis, MO, USA), 1×DreamTaq buffer (Fermentas), and 250 nM each fD1 and rP2 primers for the bacterial 16S rRNA gene [5] or 400 nM Arc8f and Arc1492r primers for the archaeal 16S rRNA gene [13]. The primer pairs used produced nearly full-length 16S rRNA gene fragments at 1495 base pairs (bp) and 1361 bp for bacteria and archaea, respectively. Thermal cycling was carried out in a Techne TC-412 thermal cycler (Techne, Burlington, NJ, USA). Conditions were 94°C for 4 min, followed by 35 cycles of 94°C for 1 min, annealing at 46°C for 1 min, and extension at 72°C for 2 min, with a final extension at 72°C for 10 min for bacterial amplification. Conditions were similar for archaeal, but with an annealing temperature at 55°C. Clones containing the 16S rRNA gene sequences were generated using the TOPO TA Cloning® Kit for Sequencing and pCR4-TOPO vector (Life Technologies) following manufacturer’s instructions. Cloned 16S rRNA gene sequences were PCR amplified with 2 nM each M13 forward and reverse primers **(** **Table 1** **)** as described above, treated with ExoSAP-IT (Affymetrix Inc, Santa Clara, CA, USA), and sequenced using an ABI 3730 capillary sequencer (Oklahoma Medical Research Foundation, Oklahoma City, OK, USA). Glycerol stocks were generated for the 16S rRNA gene clones with greater than 99% coverage and 99% maximum identity to *Thermovirga* (bacteria) or *Archaeoglobus* (archaea) type strains. Supercoiled plasmid DNA was purified from 3 ml cultures using the Geneaid plasmid mini kit (Geneaid, Agoura Hills, CA, USA). Plasmid lengths were 5451 bp and 5317 bp for bacteria and archaea, respectively. Plasmid DNA concentrations were quantified as described above and used immediately.

**Table 1:** List of primers used to amplify the 16S rRNA gene.

Primer	Direction	Taxa/Domain	Sequence (5′-3′)	Reference
*Cloning*				
fD1	Forward	Bacteria	AGAGTTTGATCCTGGCTCAG	[5]
rP2	Reverse		ACGGCTACCTTGTTACGACTT	
Arc8f	Forward	Archaea	TCCGGTTGATCCTGCC	[13]
Arc1492r	Reverse		GGCTACCTTGTTACGACTT	
*qPCR*				
27f	Forward	Bacteria	AGAGTTTGATCMTGGCTCAG	[14]
338r	Reverse		GCTGCCTCCCGTAGGAGT	
Arc8f	Forward	Archaea	TCCGGTTGATCCTGCC	[15]
Arc344r	Reverse		TCGCGCCTGCTGCICCCCGT	

### Preparation of 16S rRNA Gene Plasmid and Amplicon DNA Standards

Circular plasmid (supercoiled and nicked-circles), linearized plasmid, and PCR amplicon standards were prepared for microbial 16S rRNA gene estimate comparisons. The pCR4-TOPO vector contains three *Nb.BstI* restriction sites, therefore nicked circles were prepared by digesting 1 µg of supercoiled plasmid DNA with *Nb.BstI* restriction enzyme (New England Biolabs, Ipswich, MA, USA) according to manufacturer’s instructions for 2 hours at 37°C. Reactions were heat inactivated at 80°C for 20 min. Linearized plasmids were prepared by digesting 1 µg of supercoiled DNA with *SpeI* restriction enzyme (Promega, Madison, WI, USA) following manufacturer’s instructions for 2 hours at 37°C. Reactions were heat inactivated at 65°C for 15 min and digests were purified using the Amicon Ultra 30K filtration devices (Millipore) following manufacturer’s instructions. The completeness of the digestions was confirmed by agarose gel electrophoresis. Purified digested plasmid DNA concentrations were quantified by fluorometry as described above.

Amplicon DNA was prepared by end-point PCR targeting the V1–V2 region of 16S rRNA gene using bacterial [14] and archaeal [15] specific primer sets to generate partial 16S rRNA gene sequences of 347 bp and 356 bp, respectively. Briefly, 25 µl reactions were set up as described above containing 2 µl supercoiled *T. lienii* plasmid DNA and 250 nM 27f and 125 nM 338r primers or 2 µl *A. fulgidus* plasmid DNA and 500 nM A8f and 1 µM 344r primers. Cycling conditions were: 95°C for 5 min, 35 cycles of 95°C for 30 s, 55°C for 45 s, 72°C for 45 s, and a final extension at 72°C for 10 min. Amplicon size was verified by agarose gel electrophoresis and amplicon DNA was purified and quantified as described above.

Molar concentrations for circular, linear, and amplicon standard DNA were converted into 16S rRNA gene copies µl^−1^ based on the following assumptions: the average molecular mass of a dsDNA bp is 6.6×10^11^ ng mol^−1^, Avogadro’s number of copies mol^−1^ is 6.022×10^23^ [16]:
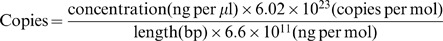



Serial 10-fold dilutions spanning from 10^7^ to 10^2^ copies µl^−1^ were generated for each type of standard using RT-PCR grade water and were used immediately.

### 16S rRNA Gene qPCR Assays

Estimates of the number of 16S rRNA gene copies in 1∶10, 1∶50, and 1∶100 dilutions of DNA from *D. vulgaris*, *P. aeruginosa*, *A. fulgidus*, and *M. jannaschii* were made using qPCR. Briefly, 30 µl reactions contained 15 µl of 2×SYBR®Green PCR Master Mix (Life Technologies), 0.5 M Betaine (Sigma-Aldrich), and V1–V2 specific 16S rRNA gene primers as described in Hamady et al. [17]. Standard DNA dilution series were assayed in triplicate, and genomic DNA samples were assayed at three dilutions (1∶10, 1∶50, and 1∶100), each in triplicate. Thermal cycling, data acquisition and analyses were carried out with the StepOnePlus™ Real-Time PCR System and StepOne Software v2.1 (Life Technologies). Cycling conditions were: 95°C for 10 min followed by 40 cycles of 95°C for 30 s, 55°C for 45 s, 72°C for 45 s, and ended with a melt curve analysis to ensure primer-dimer was excluded from the analysis. Image capture was at 72°C.

### Analysis of Standard Curves and Estimated Partial 16S rRNA Gene Copies

The amplification efficiencies for circular and linear standard curves were calculated by the StepOnePlus™ Real-Time PCR System and StepOne Software v2.1 (Life Technologies). To determine if the bacterial (or archaeal) curves were significantly different from one another, the linear regression between Ct values versus log_10_ copies for each of four sets of standards was analyzed and compared to one another using the one-way analysis of variation (ANOVA) and the Bonferroni’s multiple comparisons test in GraphPad Prism5 software (GraphPad Software, San Diego, CA, USA).

The predicted number of 16S rRNA gene copies in each dilution of *D. vulgaris*: genome size of 3,570,858 bp at 5 copies of the 16S rRNA gene sequence genome^−1^ (NC_002937), *P. aeruginosa*: genome size of 6,264,404 bp at 4 copies genome^−1^ (NC_002516), *A. fulgidus*: genome size of 2,178,400 bp at 1 copy genome^−1^ (NC_000917), and *M. jannaschii*: genome size of 1,664,970 bp at 2 copies genome^−1^ (NC_000909) was calculated based on the number of 16S rRNA gene copies ng^−1^ genomic DNA found at the *rRNA database homepage*: http://rrndb.mmg.msu.edu/search.php. Genome size was converted into ng using the following equation:
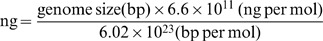



Estimated 16S rRNA gene copies based on the circular and linear standard curves were compared to the number of predicted copies and the ratio was used to assess the degree of inflation (or reduction) based on each of the standard DNA conformations.

## Results

### Comparison of Standard Curves

**Figure 1:**
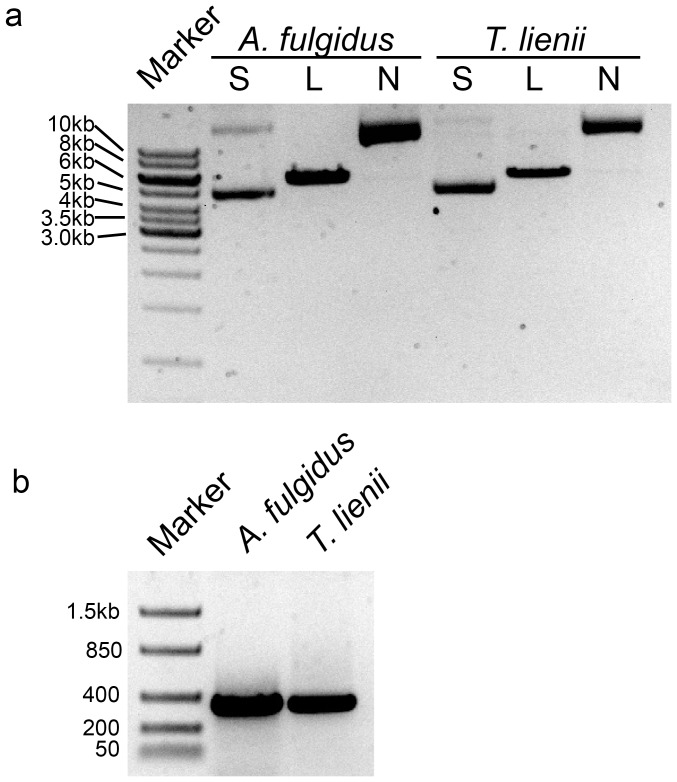
Preparation of 16S rRNA gene standards. Representative archaeal (*A. fulgidus*) and bacterial (*T. lienii*) (**a**) plasmids: Marker = 1 kb DNA ladder, S = freshly isolated supercoiled plasmid, L = linearized plasmid (*SpeI*-digested), and N = nicked circular plasmid (*Nb.BtsI*-digested) and (**b**) PCR amplicons: Marker = low range DNA ladder.

Plasmid DNA is routinely used to generate standards for qPCR analysis and exists primarily in the circular form [9]. A recent report suggested that linearized plasmids were more accurate at quantifying gene estimates in eukaryotic genomes [7]. Therefore, we sought to compare two conformations of circular DNA and two linearized DNA standards in estimating numbers of 16S rRNA gene copies in genomic DNA samples from microbial strains with sequenced genomes. First, nicked circles and linearized bacterial (*T. lienii*) and archaeal (*A. fulgidus*) 16S rRNA gene plasmids and PCR amplicons were prepared from supercoiled plasmid DNA by *Nb.BtsI* digest, *SpeI* digest, and end-point PCR, respectively. The four DNA preparations were purified, quantified using Qubit fluorometry, and analyzed by agarose gel electrophoresis **(** **Figure 1** **)**. Propagated plasmids isolated from transformed bacterial cells were predominantly supercoiled DNAs that ran faster than their linearized counterparts **(** **Figure 1a** **,** ***compare lanes labeled S to lanes L*** **)**, whereas the nicked circles ran much slower than both the linearized and supercoiled plasmids. The 16S rRNA gene amplicons that spanned the V1–V2 region were approximately 350 base pairs in length **(** **Figure 1b** **.)**.

**Figure 2:**
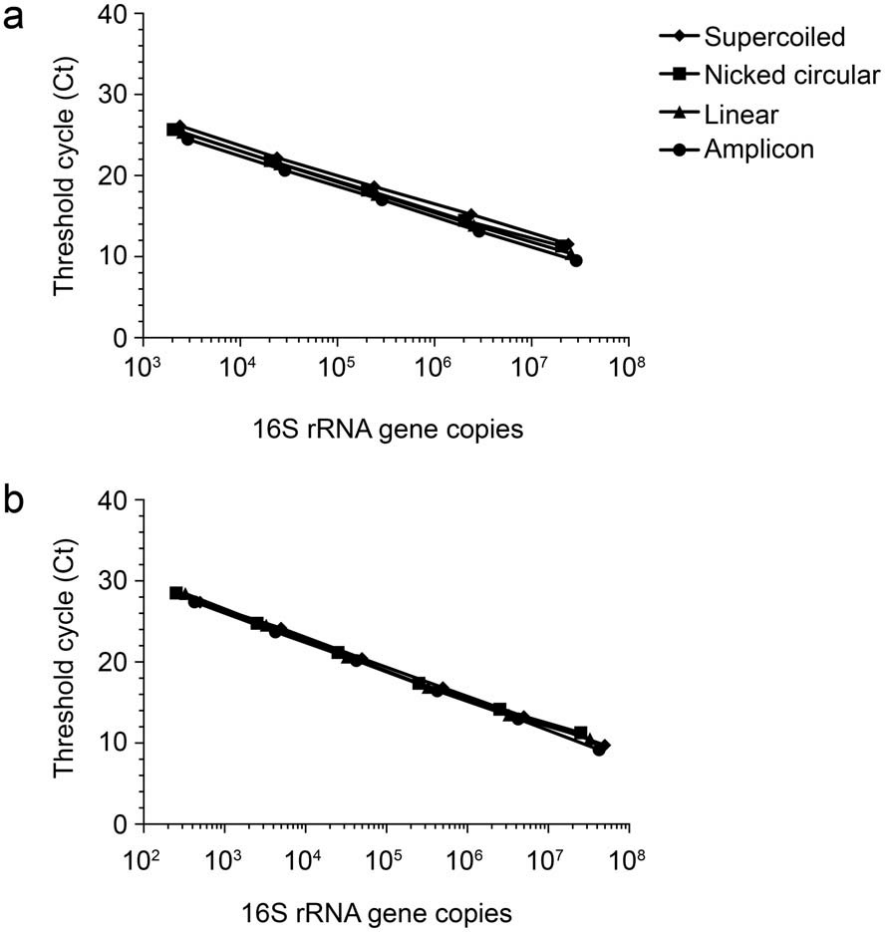
Circular and linear standard curves have similar slopes, y-intercepts, and amplification efficiencies. Linear regression between log_10_ 16S rRNA gene copies and Ct value based on (**a**) bacterial (*T. lienii*) and (**b**) archaeal (*A. fulgidus*) standards. Diamonds = supercoiled plasmid standard, squares = nicked-circular plasmid standard, triangles = linearized plasmid standard, and circles = PCR amplicon-based standard. Data are the average (n = 3) and error bars are ±1 standard deviation between replicates. Slopes and y-intercepts were not significantly different for bacterial (P = 0.97) or archaeal (P = 0.99) curves.

Next, to determine if the conformation of the DNA standard significantly affected amplification efficiency, the performance of qPCR reactions using serial dilutions of the four prepared standards were compared **(** **Figure 2** **)**. Bacterial *T. lienii* curves spanned from 10^7^ to 10^3^ copies **(** **Figure 2a** **and** **Table 2** **)** and the performance of each standard curve is summarized in **Table 3**. Amplification efficiencies ranged from 85% to 89%, and an ANOVA showed that there was no significant difference between the slopes or y-intercepts of the four curves (P = 0.97).

**Table 2:** Standard setup and Ct range for qPCR reactions.

16S	Standard type	Standard range	Ct value range	NTC Ct^a^
Bacteria^b^	Amplicon	2.89×10^3^ to 2.89×10^7^	9.50±0.49 to 24.48±0.06	≥35
	Linear	2.56×10^3^ to 2.56×10^7^	10.39±0.14 to 25.35±0.11	≥35
	Nicked circles	2.04×10^3^ to 2.04×10^7^	11.31±0.03 to 25.68±0.17	≥35
	Supercoiled	2.40×10^3^ to 2.40×10^7^	11.53±0.07 to 26.17±0.03	≥35
Archaea^c^	Amplicon	4.25×10^2^ to 4.25×10^7^	9.18±0.10 to 27.43±0.17	≥35
	Linear	3.28×10^2^ to 3.28×10^7^	10.54±0.02 to 28.46±0.01	≥35
	Nicked circles	2.52×10^2^ to 2.52×10^7^	11.27±0.15 to 28.52±0.15	≥35
	Supercoiled	4.99×10^2^ to 4.99×10^7^	9.74±0.19 to 27.39±0.11	≥35

a Ct value for the NTC (no template control).

b Bacteria: *T. lienii.*

c Archaea: *A. fulgidus.*

**Table 3:** Performance of microbial standard DNA in qPCR reactions.

16S	Standardtype	Standard curve^a^	R^2^	Efficiency (%)^b^
Bacteria^c^	Amplicon	y = −3.74±0.03x +37.41±0.21	0.999	84.9
	Linear	y = −3.75±0.02x +38.05±0.14	0.999	84.7
	Nicked circles	y = −3.61±0.04x +37.49±0.20	0.999	87.3
	Supercoiled	y = −3.63±0.03x +38.26±0.15	0.999	88.7
Archaea^d^	Amplicon	y = −3.64±0.01x +36.99±0.07	0.999	88.4
	Linear	y = −3.62±0.06x +37.22±0.32	0.999	89.0
	Nicked circles	y = −3.48±0.06x +36.62±0.31	0.999	93.7
	Supercoiled	y = −3.56±0.02x +37.15±0.13	0.999	91.0

a Linear regression between Ct (y-intercept) and log_10_ starting copy number (x, i.e. slope).

b Efficiency (%) calculated: E = (10^1/slope^−1)×100.

c Bacteria: *T. lienii.*

d Archaea: *A. fulgidus.*

Similar results were obtained for the *A. fulgidus* 16S rRNA gene standards **(** **Figure 2b** **and** **Table 2** **and** **Table 3** **)**. Amplification efficiencies ranged from 88% to 94% and the four curves were not significantly different from one another (P = 0.99) by ANOVA. Therefore, the conformation of the standard had a negligible effect on the performance of the qPCR reactions. These results were not unexpected, as the efficiencies were not consistently different for eukaryotic gene amplification [7].

### Comparison of Microbial 16S rRNA Gene Copies Based on Standard Curves

While Hou et al. [7] found no consistent difference between amplification efficiencies between circular and linear curves, they did however find that standard curves based on the circular plasmids overestimated the number of gene copies in their eukaryotic system by approximately 8-fold. Therefore, using two bacterial and two archaeal genomes we asked if either circular plasmid conformation caused the same degree of inflation. Genomic DNA samples were assayed at three dilutions: 1∶10, 1∶50, and 1∶100, each in triplicate. This range was deemed appropriate as DNA extracted from environmental samples may contain inhibitors to the qPCR reaction in the DNA preparations at stock concentration reviewed in [18].

**Figure 3:**
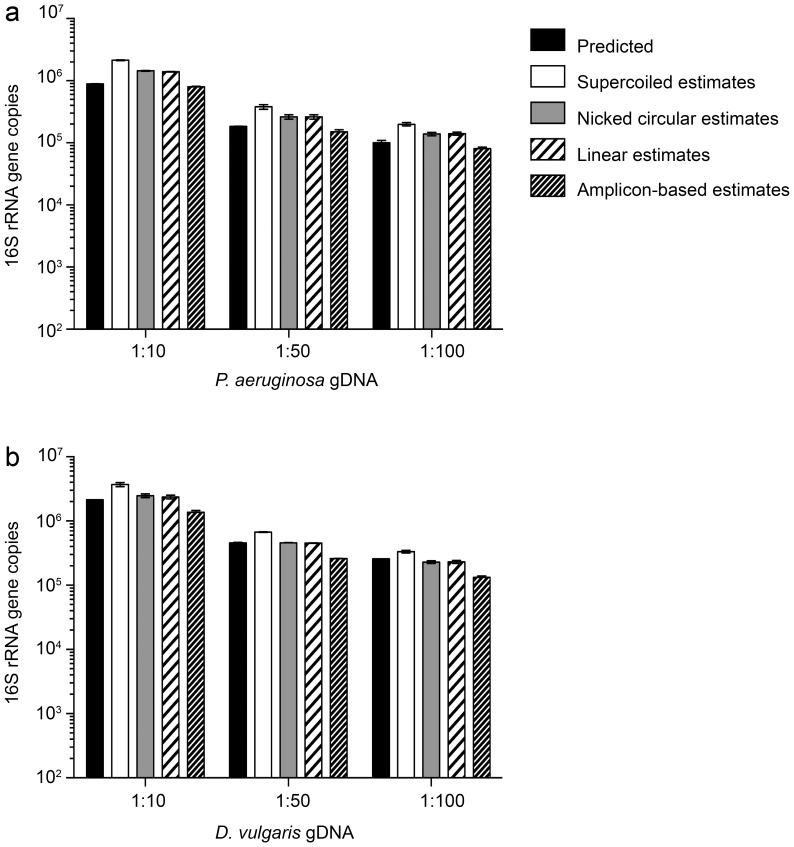
Comparison of expected and estimated 16S rRNA gene copies in bacterial DNA samples. Expected bacterial 16S rRNA gene copies were calculated based on four and five 16S copies per genome for (**a**) *P. aeruginosa* and (**b**) *D. vulgaris*, respectively. Black bars = predicted 16S copies. White bars = estimated 16S copies based on supercoiled plasmid standard. Grey bars = estimated 16S copies based on nicked-circular plasmid standard. Black and white striped bars = estimated 16S copies based on linearized plasmid standard. Black and gray striped bars = estimated 16S copies based on amplicon-based standard. Data are the average (n = 3) and error bars are ±1 standard deviation among replicates.

**Figure 4:**
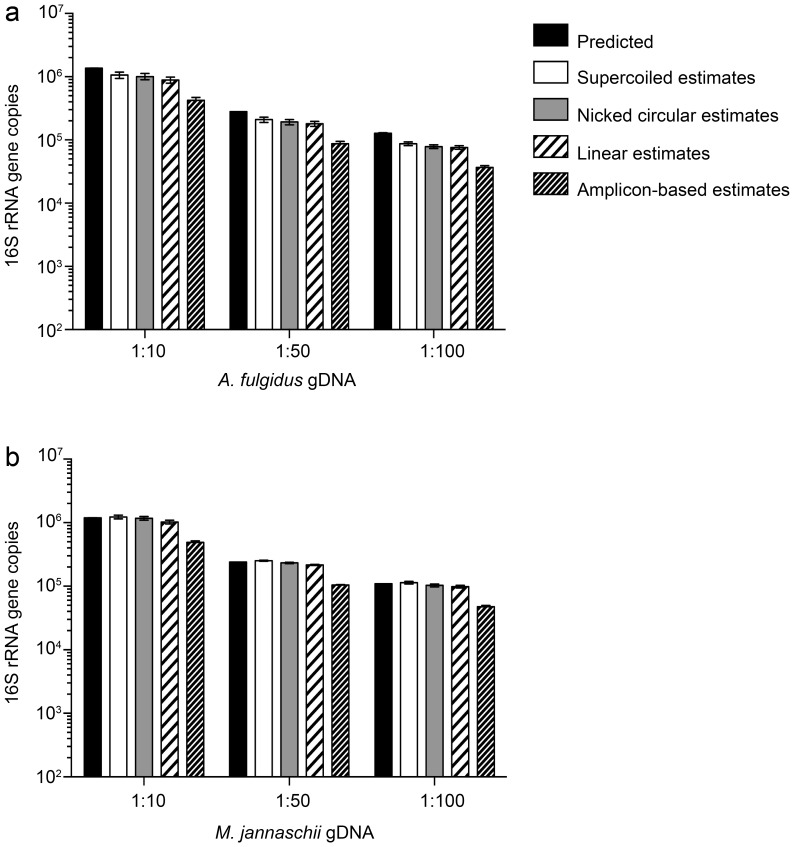
Comparison of expected and estimated 16S rRNA gene copies in archaeal DNA samples. Expected archaeal 16S rRNA gene copies were calculated based on one and two 16S copies per genome for (**a**) *A. fulgidus* and (**b**) *M. jannaschii*, respectively. Black bars = predicted 16S copies. White bars = estimated 16S copies based on supercoiled plasmid standard. Grey bars = estimated 16S copies based on nicked circular plasmid standard. Black and white striped bars = estimated 16S copies based on linearized plasmid standard. Black and gray striped bars = estimated 16S copies based on amplicon standard. Data shown are representative of two experiments. Data are the average (n = 3) and error bars are ±1 standard deviation among replicates.

The estimated number of bacterial 16S rRNA gene copies, based on the four standard curves, was compared to predicted 16S rRNA gene copy numbers **(** **Figure 3** **and** **Table 4** **)**. For both bacterial genomes, gene estimates derived from nicked circles and linearized plasmids were indistinguishable from one another. For both archaeal genomes, estimates derived from both linear and circular standard curves approached 1 **(** **Figure 4** **and** **Table 4** **)**. Note that the *A. fulgidus* 16S rRNA gene sequence was used as the standard for the archaeal qPCR reactions and was expected to be a precise match. Interestingly, both circular plasmids provided the best estimates for the archaeal 16S rRNA gene. Taken together, these results demonstrate than no single standard conformation performed the best in all instances. Importantly, estimates using the supercoiled standard never approached the 8-fold overestimates noted for eukaryotic systems.

**Table 4:** Estimated and expected 16S rRNA gene copies in microbial gDNA samples based on qPCR standard curves.

Genome	Type	1∶10	1∶50	1∶100	Ratio^a^
*P. aeruginosa*	Amplicon	8.01±0.11×10^5^	1.51±0.12×10^5^	8.08±0.48×10^4^	0.84±0.06
	Linear	1.39±0.02×10^6^	2.62±0.21×10^5^	1.41±0.08×10^5^	1.42±0.51
	Nicked circles	1.44±0.02×10^6^	2.62±0.22×10^5^	1.39±0.08×10^5^	1.48±0.52
	Supercoiled	2.14±0.03×10^6^	3.80±0.32×10^5^	2.00±0.12×10^5^	2.17±0.76
	**Predicted** b	**8.87±0.10×10^5^**	**1.84±0.01×10^5^**	**1.01±0.08×10^5^**	
*D. vulgaris*	Amplicon	1.36±0.09×10^6^	2.61±0.02×10^5^	1.33±0.06×10^5^	0.50±0.19
	Linear	2.36±0.16×10^6^	4.53±0.03×10^5^	2.31±0.11×10^5^	0.87±0.34
	Nicked circles	2.48±0.17×10^6^	4.59±0.03×10^5^	2.30±0.14×10^5^	0.89±0.35
	Supercoiled	3.70±0.26×10^6^	6.71±0.04×10^5^	3.34±0.17×10^5^	1.30±0.52
	**Predicted**	**2.15**±**0.04x×10^6^**	**4.60±0.04×10^5^**	**2.58±0.01×10^5^**	
*A. fulgidus*	Amplicon	7.04±0.76×10^5^	1.44±0.13×10^5^	6.11±0.39×10^4^	0.50±0.04
	Linear	8.87±0.92×10^5^	1.80±0.17×10^5^	7.60±0.49×10^4^	0.63±0.06
	Nicked circles	1.01±0.11×10^6^	1.92±0.18×10^5^	7.86±0.53×10^4^	0.68±0.08
	Supercoiled	1.06±0.11×10^6^	2.10±0.20×10^5^	8.72±0.56×10^4^	0.74±0.07
	**Predicted**	**1.36±0.05×10^6^**	**2.80±0.01×10^5^**	**1.28±0.02×10^5^**	
*M. jannaschii*	Amplicon	8.13±0.51×10^5^	1.73±0.04×10^5^	7.92±0.36×10^4^	0.71±0.03
	Linear	1.03±0.06×10^6^	2.16±0.04×10^5^	9.86±0.45×10^4^	0.89±0.04
	Nicked circles	1.17±0.08×10^6^	2.33±0.05×10^5^	1.03±0.05×10^5^	0.96±0.04
	Supercoiled	1.23±0.08×10^6^	2.52±0.06×10^5^	1.14±0.05×10^5^	1.04±0.04
	**Predicted**	**1.19±0.01×10^6^**	**2.41±0.01×10^5^**	**1.10±0.01×10^5^**	

a Ratio of estimated divided by predicted 16S copies averaged across the three dilutions.

b Predicted copies calculated as described in **Methods**.

## Discussion

Propagated plasmid DNA containing a gene sequence of interest is likely the most common form used to generate standards for the quantitative analysis of gene copies [19] due to its ease of preparation. In most instances the form of the standard is not reported and only recently has it come into question. A recent study [7] compared the precision of gene estimates in eukaryotic systems based on linear versus circular standards, but this effect of the conformation of the DNA standard was only tested in eukaryotic systems. It was concluded that supercoiled plasmids led to approximately 8-fold overestimates relative to its linearized counterpart and suggested that these findings be tested in systems whose target DNA is itself circular [7]. Therefore, the goal of this study was to determine if circular plasmids led to similar overestimates using representatives from microbial domains. We demonstrated that estimates of 16S rRNA gene copies did not approach the 8-fold overestimates reported for eukaryotic systems [7]. Indeed, estimates derived from the supercoiled standard curves ranged from 0.5 to 2.2-fold and no single conformation provided the best estimates for the genomes tested.

Aside from the conformation of the DNA target gene, several variables between the three studies could account in part for the differences in magnitude of gene estimates observed between eukaryotic versus prokaryotic systems. Those include but are not limited to: 1) the conformation of the circular standard tested and 2) the preparation of the standards. In the Hou et al. study [7], the implication that the circular plasmid was supercoiled must be inferred from the text, as a gel image was not included. In any case, estimates were much higher than those using the linear standard. In the maize study [8], ≥3-fold inflation in gene estimates was observed for the supercoiled versus the linearized standard. Interestingly, both supercoiled and nicked circular plasmids were prepared, but only the supercoiled was investigated for its affect on estimates using genomic DNA [8]. In results presented here, the effect of both circular plasmid conformations were assayed using microbial genomic DNA. Another source of variation was the method of standard preparation and quantification. Hou et al. purified the plasmids and amplicons prior to quantification based on the optical absorbance at OD_260_ [7]. Lin et al. demonstrated that supercoiled and linearized DNA showed differences in quantification based on the optical absorbance, but took measurements prior to purification [8]. In the present study, the digested plasmid standards and amplicons were purified and quantified following digestion or amplification to rid of enzymes or other contaminants that could interfere with quantification readings or compete with components in the qPCR reaction.

Our objective was to determine if a propagated plasmid DNA standard was suitable for prokaryotic gene estimates where qPCR analyses are performed on a routine basis. Little standard preparation is required for using propagated plasmids aside from quantifying and diluting a frozen plasmid aliquot prior to qPCR setup. Minimal preparation of standards, in lieu of linearization or PCR amplification and purification saves time and reagents and gives the same quality data as the more time-consuming standard preparation methods. We therefore believe our results showing similar estimates of the 16S rRNA gene copy number support the use of circular plasmids for qPCR standards. Circular plasmid standards will facilitate the practical analysis of industrial and environmental samples in labs that perform many different qPCR assays targeting different microbial taxa.
